# Sex and nest type influence avian blood parasite prevalence in a high-elevation bird community

**DOI:** 10.1186/s13071-021-04612-w

**Published:** 2021-03-08

**Authors:** Marina D. Rodriguez, Paul F. Doherty, Antoinette J. Piaggio, Kathryn P. Huyvaert

**Affiliations:** 1 Department of Fish, Wildlife and Conservation Biology, Colorado State University, Fort Collins, Colorado USA; 2 National Wildlife Research Center, U.S. Department of Agriculture, Fort Collins, Colorado USA

**Keywords:** Haemosporidia, *Haemoproteus*, *Plasmodium*, Occupancy modeling

## Abstract

**Background:**

The prevalence of avian haemosporidian parasites and the factors influencing infection in the Colorado Rocky Mountains are largely unknown. With climate change expected to promote the expansion of vector and avian blood parasite distributions, baseline knowledge and continued monitoring of the prevalence and diversity of these parasites is needed.

**Methods:**

Using an occupancy modeling framework, we conducted a survey of haemosporidian parasite species infecting an avian community in the Colorado Rocky Mountains in order to estimate the prevalence and diversity of blood parasites and to investigate species-level and individual-level characteristics that may influence infection.

**Results:**

We estimated the prevalence and diversity of avian Haemosporidia across 24 bird species, detecting 39 parasite haplotypes. We found that open-cup nesters have higher *Haemoproteus* prevalence than cavity or ground nesters. Additionally, we found that male Ruby-crowned Kinglets, White-crowned Sparrows, and Wilson’s Warblers have higher *Haemoproteus* prevalence compared to other host species. *Plasmodium* prevalence was relatively low (5%), consistent with the idea that competent vectors may be rare at high altitudes.

**Conclusions:**

Our study presents baseline knowledge of haemosporidian parasite presence, prevalence, and diversity among avian species in the Colorado Rocky Mountains and adds to our knowledge of host–parasite relationships of blood parasites and their avian hosts.
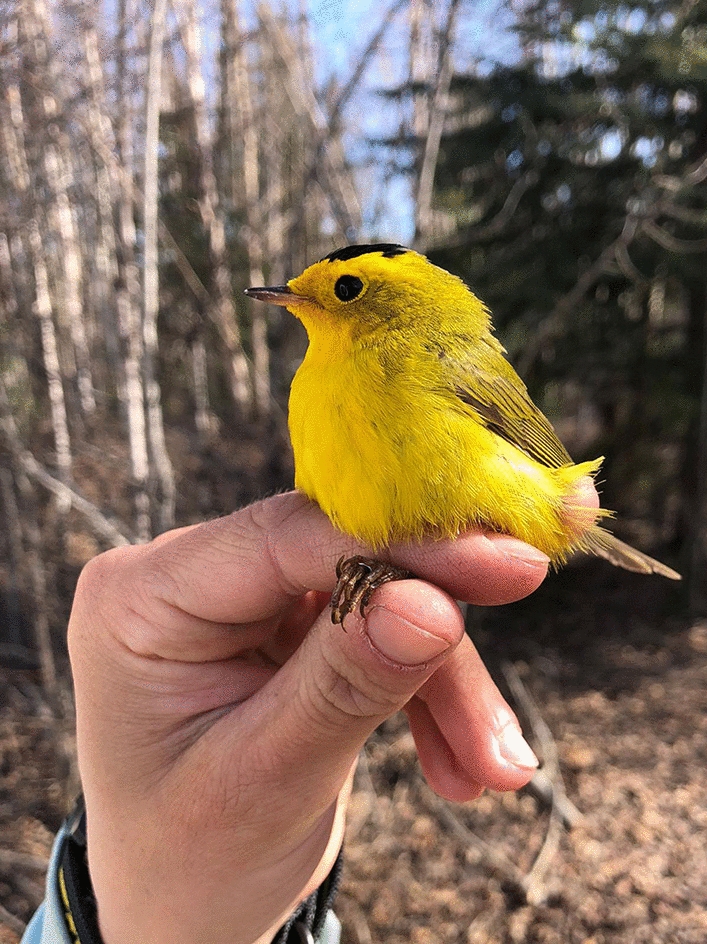

## Background

Parasitism is an important driver of ecological and evolutionary processes [1, 2] as parasites may regulate host population size, e.g., Hochachka and Dhondt [3], affect species interactions, e.g., Ricklefs [4], and create selection pressures in wild populations, e.g., Laine [5]. Compounding effects of parasites with other factors such as climate change, competition with invasive species, habitat loss, or harsh environmental conditions can also drive populations to low numbers, predisposing them to local or global extinction [6–9].

Haemosporida (Phylum: Apicomplexa) are protozoan parasites that infect the blood cells of vertebrates and are transmitted by dipteran vectors [10]. These blood parasites—haemosporidian parasites—are distributed worldwide and infect a number of vertebrates, including mammals [11], reptiles [12], and birds [10]. Blood parasites go through sexual reproduction in dipteran vectors and are transmitted to vertebrate hosts during vectors’ blood meals [10]. Once in a competent host, the parasite undergoes asexual reproduction and the infected host becomes a reservoir, carrying developed gametocytes within its red blood cells [10].

Among vertebrates, birds are hosts to the highest diversity of haemosporidian parasites, with records of birds being infected with over 200 morphologically distinct haemosporidian parasite species [10, 13, 14] and over 3000 unique haplotypes [13]. The three parasite genera that infect birds include *Haemoproteus*, *Plasmodium*, and *Leucocytozoon* [10 and 15]. Negative effects of infection can be due to changes in host behavior [16] or to severe physiological responses, resulting in high mortality rates during the acute phase of infection [17, 18]. Avian hosts can also suffer declines in reproductive success [19, 20] and reduced lifespan when enduring chronic infections [21]. Within species, factors such as age, sex, immune status, and degree of exposure may also contribute to variation in host susceptibility and mortality [15].

Spatial and temporal dynamics of avian haemosporidian parasite occurrence are governed by environmental, ecological, and demographic characteristics [22–25]. In temperate environments, seasonality has a strong influence on the survival and development of both parasites and insect vectors, as mosquitoes emerge during the spring and are active until the end of the summer [26]. This increase in parasites and vectors coincides with the breeding season for most avian species, when resource allocation is diverted to reproduction instead of immune function [27, 28]. Our study took place during the breeding season, allowing us to survey avian blood parasites at a time when infection frequency is expected to be highest.

The intensity and seasonality of haemosporidian parasite transmission tends to vary by elevation [29]. Negative correlations between elevation and abundance of mosquitos, the main vectors of avian blood parasites, have been found in many systems including the mountains in Colorado’s Front Range [30, 31], where our study site is located. Many parasites have elevation limits because of the constraints of lower ambient temperatures encountered at higher elevations, though distributions are expanding with climate change, as the transmission of most vector-borne parasites may be enhanced by higher ambient temperatures, e.g. Samuel et al. [32], La Pointe et al. [33]. Changes in environmental conditions for vectors, such as an increase in mean air temperature and declining precipitation, support the expansion of haemosporidian parasites into habitats where lower temperatures previously limited transmission [34, 35]. For example, *Culex tarsalis* and *C. pipiens* are important vectors of *Plasmodium* parasites at lower elevations in northeastern Colorado, but the low abundance of these species at higher elevations may mean that *Plasmodium* is not yet established in areas such as Rocky Mountain National Park [36]. However, little research has been done on the distribution of haemosporidian parasites in Colorado, especially in high-elevation communities like those in the Colorado Rockies.

Across species, factors such as nest type and migration strategy may explain variation in host susceptibility. Although differences in parasite prevalence across nest types is not always found [37–39], open-cup nesting has been linked to higher blood parasite prevalence in numerous studies due to higher vector exposure for incubating individuals compared to cavity- or ground-nesting birds [40–44]. Migration has important implications for the emergence and spread of infectious disease-causing parasites due to long-distance movements and exposure to diverse habitats of infected hosts. Establishment of parasites and expansion of their ranges may take place through migration of host species, as parasites are able to survive at higher elevations as environmental conditions become more suitable for parasites and vectors [45]. Migratory birds can harbor high-intensity infections and are host to biologically diverse haemosporidian parasite species [4], allowing them to act as a source of infection to non-migratory birds who may be more susceptible to blood parasites due to lack of previous exposure at higher elevations [46, 47]. With the potential for migratory birds to spread avian blood parasites to new areas, and a warming climate allowing for the spread of blood parasites into new environments, parasite surveillance is needed in bird communities, even those previously thought to be in areas with low parasite prevalence.

Parasite surveys can serve as early indicators of disease outbreaks that could affect the health of avian populations. The study of blood parasites requires knowledge of baseline levels of haemosporidian parasite infection in target host populations to aid in the detection of temporal shifts of parasite diversity to evaluate changes in the prevalence of infection. Using an occupancy approach, we conducted multiple screenings for blood parasites per host in order to better estimate the detection probability of haemosporidian parasites within host species [48, 49]. Occupancy modeling approaches are useful in wildlife disease ecology because they acknowledge that uncertainty, such as false-negative results, exists when using imperfect diagnostic tests [50, 51]. Variation in detection among multiple screenings of the same blood sample supports the need to use an occupancy modeling framework in order to take detection probability into account in wildlife disease studies [49].

The objectives of our study were (1) to obtain baseline prevalence and diversity estimates for an avian community in the Colorado Front Range Rocky Mountains, where avian parasite surveys have not taken place, and (2) to test differences in prevalence and diversity for various individual and species characteristics. Our hypotheses related to the second objective regarding specific host and environmental predictor variables are presented in Table 1.

**Table 1:** Predicted associations, and explanations, of each predictor variable with haemosporidian parasite prevalence and detection probability

Parameter(s)	Predictor variable	Predicted direction	Explanation
Occupancy probability (ψ)	Age	Q	For adult birds, prevalence of blood parasites decreases with age due to increased immunocompetence, until old age, in which immunosenescence occurs and is linked to a higher probability of infection [85]
	Sex (female)	+	The cost of reproduction is higher in females, decreasing immunocompetence and making females more susceptible to haemosporidian infection [86].
	BCI [mass (g): tarsus length (mm)]	−	Individuals in good condition have stronger immunocompetence and are better able to fight infections [89, 88]
	Migration status	+	The energetic cost of migration increases with distance, and likely affects immunocompetence [90].
	Nest type (open nest)	+	Open nests are more exposed to mosquito vectors compared to closed nests [91]
Detection probability (*p*)	PCR run	+	Differences in results of PCR runs may occur due to variation in parasitemia between samples, with higher parasitemia associated with higher probability of detection [92]

Positive (+), negative (−), or quadratic (Q) predicted associations, and explanation, of each predictor variable for haemosporidian parasite occupancy probability (ψ; prevalence) and detection probability (*p*). BCI is body condition index defined as body mass:tarsus length. PCR is polymerase chain reaction

## Methods

### Study system

Our study area was located at the Colorado State University Mountain Campus in Larimer County, Colorado, USA (N40.5611, W105.5978), within a mountain valley at an elevation of 2750 m. The valley is a breeding site for numerous bird species, and no prior research on avian blood parasites has been conducted there to our knowledge.

### Data collection

We collected data during the summers of 2017 and 2018. The field portion of our study began in early June, when birds begin breeding, and continued through the end of the breeding season, around mid-August. We captured birds using mist nets set in sites with high passerine activity. Netting sites were in riparian, forested, and edge habitats. Song playbacks were used to attract birds to nets, providing larger sample sizes to facilitate comparisons of parasite prevalence across host species.

Captured birds were identified at the species level and banded. Sex and age were determined when possible based on guidelines from Pyle [52], morphological measurements were taken (tarsus length (mm), wing chord (mm), mass (g)), and 10–20 µl of blood was collected by brachial venipuncture and stored on Nobuto Blood Filter Strips for later DNA extraction. All birds were handled and sampled under a federal bird banding permit from the US Geological Survey (USGS) Bird Banding Laboratory and in accordance with approved guidelines of the Institutional Animal Care and Use Committee of Colorado State University (Protocol 17-7309A).

### DNA extraction, PCR amplification, and sequencing

We assessed haemosporidian parasite infection prevalence and parasite diversity using molecular techniques. We took a 2 mm hole punch of blood-soaked Nobuto blood filter strip (with ~15 µL of blood) for each bird and extracted DNA using the DNeasy 96 Blood & Tissue Kit (QIAGEN, Valencia, CA), following the manufacturer’s dried blood spot protocol. We stored extracted DNA at −20 °C prior to screening. We screened an aliquot of DNA for parasite presence using a nested polymerase chain reaction (PCR) protocol to amplify a segment of mitochondrial DNA (mtDNA) from the cytochrome *b* gene as outlined in Hellgren et al. [53]. Primers HaemNF1 and HaemNR3 were used to amplify an initial 617-bp segment of mtDNA from species of haemosporidian parasites. The conditions for this PCR were as follows: 30 seconds at 94 °C, 30 seconds at 50 °C, and 45 seconds at 72 °C for 20 cycles. The samples were incubated before the cyclic reaction at 94 °C for 3 minutes and after the cyclic reaction at 72 °C for 10 minutes. An aliquot of the product (1 µL) from the first PCR reaction was used in a second reaction amplifying a 479-bp segment of *Haemoproteus* and *Plasmodium* lineages using primers HaemF and HaemR2 [53]. The conditions for the second round of PCR assays are as follows: 30 seconds at 50 °C, and 45 seconds at 72 °C for 35 cycles. PCR screening was carried out three times for each sample, and each plate (96 samples) included two positive controls (one for *Haemoproteus* and one for *Plasmodium*) and one negative control. All PCR reactions were performed at a final volume of 25 µl using illustra PuReTaq Ready-To-Go™ beads (GE Healthcare) with freeze-dried, pre-formulated reagents. We ran 5 µl of the final product on a 2% agarose gel to screen for parasite presence. For host individuals infected with haemosporidian parasites, the final PCR product was cleaned with ExoSAP-IT (Thermo Fisher Scientific) prior to sequencing.

The sequencing reaction of 10 µl contained 0.25 µl BigDye™ (Thermo Fisher Scientific), 2.275 μl BigDye™, 1 µl of each nested secondary PCR primer (HaemF and HaemR2), 1 µl of PCR product, and 5.475 µl molecular-grade ddH2O. Cycle sequencing was conducted at 94 °C for 2 min; 40 cycles of amplification at 85 °C for 10 s; 53 °C for 10 s and 60 °C for 2.5 min. The sequencing reactions were cleaned up using 600 µl of Sephadex^®^ G-50 solution per sample prior to analysis on an automated Applied Biosystems (ABI) 3500 Genetic Analyzer. Forward and reverse reads were assembled and edited using Geneious Prime 2019.0.4 (https://www.geneious.com). Mixed sequences, as indicated by double peaks in a chromatogram, were considered co-infections [44]. We identified all sequences at the genus level using the Basic Local Alignment Search Tool (BLAST) feature in the MalAvi database [13] (http://mbio-serv2.mbioekol.lu.se/Malavi/), a database for avian blood parasites. Mitochondrial haplotypes, that is, sequences differing by one or more bases (<100% identity) from known parasite lineages, were considered unique lineages [53].

### Statistical analyses

We carried out each PCR assay three times for each DNA sample in order to obtain a parasite detection history composed of 1s and 0s for each individual, with 1 signifying at least one detected parasite and 0 indicating no parasite detected. With this detection history, we estimated the probability of parasite detection along with the proportion of individuals infected with blood parasites, corrected for detection probability. We analyzed detection histories for avian haemosporidian parasites using the single-season occupancy model in Program MARK [54] to estimate the prevalence (i.e., occupancy) of each genus of parasite for each bird species as well as across species [49, 55]. In a typical occupancy framework, randomly selected “sites” are surveyed on multiple occasions within a period where occupancy state is assumed not to change. Repeated survey occasions at each site allow the estimation of two parameters: occupancy (ψ), the probability that a site is occupied by the species of interest, and detection probability (p), the probability that the species is detected during a given occasion if the site is occupied [56]. In our study, each blood sample from an individual bird is analogous to a site, the species of interest are *Haemoproteus* and *Plasmodium* parasites, and the repeated survey occasions are multiple replicates of PCR assays for each DNA sample. Reinterpreting the model parameters for parasite detection gives ψ_i_ as the prevalence of a parasite infection, and *p*_i_ as the probability of detecting a parasite(s) in site *i*, given the presence of the parasite(s) in the host.

We carried out analyses for *Haemoproteus* and *Plasmodium* parasite prevalence separately. We constructed a candidate model set for an all-species analysis that included all birds captured and sampled, as well as a species-specific candidate model set for each host species with at least 20 DNA samples (10 species). In order to address any individual heterogeneity that may exist, we used the random effects model in Program MARK to incorporate any heterogeneity beyond our predictions in each model. Our model set consisted of all possible combinations of predictor variables (Table 1), and we used an information-theoretic approach for model ranking and selection [57]. We calculated Akaike weights (w_i_; the weight of evidence in favor of each model being the best model compared to the rest of the models in the set) and considered the variables with a cumulative weight greater than 0.5 to be the most important [58].

## Results

In 2017, we captured 232 birds, and in 2018 we captured 206 birds. Of the 438 birds captured, 180 were males, 206 were females, and for the remainder sex could not be determined. We captured 24 hatch-year birds, 135 second-year birds, and 241 after second-year birds, and we could not determine age in the remaining 38 birds. Body condition indices (mass:tarsus) ranged from 0.20 to 25.71 g/mm. We collected molecular data from a total of 437 birds belonging to 24 species over the 2 years of the study (Table 2).

**Table 2:** Host bird species sampled and haemosporidian lineages detected in each host species

Bird species (nest type)	# Sampled individuals	# *Plasmodium* infections detected	# *Haemoproteus* infections detected	Molecular lineages (# detected in each species)
American Robin *(Turdus migratorius; T)*	28	2	11	H_JUHYE03 (1), H_TURDUS2 (3), H_PHYBOR04 (1), H_VIGIL07 (1), H_TUMIG07 (1), H_TUMIG08 (2), H_POEATR01 (1), H_DENADE01 (1), P_LAIRI01 (2)
American Three-toed Woodpecker (*Picoides dorsalis*; C)	1	0	0	
Brown Creeper (*Certhia americana*;C)	7	0	1	H_SISKIN1 (1)
Chipping Sparrow (*Spizella passerine*; G)	2	0	1	H_VIGIL07 (1)
Cordilleran Flycatcher (*Empidonax occidentalis*; T)	20	1	1	H_PHYBOR04 (1), P_PADOM11 (1)
Dark-eyed Junco (*Junco hyemalis*; G)	31	1	6	H_VIOLI05 (2), H_SISKIN1 (3), H_TURDUS2 (1), P_WW3 (1)
Golden-crowned Kinglet (*Regulus satrapa*; T)	2	1	1	H_MELCAR01 (1), P_LAIRI01 (1)
Green-tailed Towhee (*Pipilo chlorurus*; G)	1	0	0	
Hairy Woodpecker (*Dryobates villosus*; C)	3	0	0	
Hermit Thrush (*Catharus guttatus*; G)	5	0	2	H_JUHYE03 (1), H_TUMIG07 (1)
House Wren (*Troglodytes aedon*; C)	6	0	1	H_GYMSAL01 (1)
Lincoln's Sparrow (*Melospiza lincolnii*; G)	63	4	13	H_GYMSAL01 (1), H_TURDUS2 (3), H_VIGIL07 (3), H_MELCAR01 (1), H_TUMIG08 (1), H_PHYBOR04 (1), H_DENADE01 (2), H_TUMIG06 (1), P_CATUST06 (1), P_PADOM11 (1), P_WW3 (1), P_BAEBICO2 (1)
MacGillivray's Warbler (*Geothlypis tolmiei*; G)	6	0	1	H_PHYBOR01 (1)
Mountain Chickadee (*Poecile gambeli*; C)	22	2	3	H_SISKIN1 (1), H_TURDUS2 (1), H_PHYBOR01 (1), P_WW3 (2)
Northern Flicker (*Colaptes auratus*; C)	5	0	2	H_GYMSAL01 (1), H_ZOCAP08 (1), H_SISKIN1 (1), H_MELCAR01 (1)
Pine Siskin (*Spinus pinus*; T)	45	1	8	H_TABI10 (1), H_JUHYE03 (1), H_SISKIN1 (2), H_PASILI01 (1), H_TURDUS2 (2), H_MELICAR01 (1), P_PADOM11 (1)
Red-breasted Nuthatch (*Sitta canadensis*; C)	24	0	3	H_SISKIN1 (2), H_VIGIL07 (1)
Red-naped Sapsucker (*Sphyrapicus nuchalis*; C)	6	0	1	H_TUMIG07 (1)
Ruby-crowned Kinglet (*Regulus calendula*; T)	23	0	5	H_SISKIN1 (1), H_ZOCAP08 (1), H_TURDUS2 (1), H_TUMIG06 (1), H_TUMIG07 (1), H_VIGIL07 (1)
Tree Swallow (*Tachycineta bicolor*; C)	12	0	2	H_GYMSAL01 (1), H_TUMIG07 (1)
Warbling Vireo (*Vireo gilvus*; T)	22	3	14	H_TUMIG07 (2), H_TUPHI01 (2), H_PHYBOR04 (2), H_TUMIG08 (1), H_SETAUD08 (1), H_TABIO2 (1), H_VIGIL07 (2), H_VIGIL01 (1), H_PERCAN07 (2), H_MELCAR01 (1), P_PADOM11 (2), P_BAEBICO2 (1)
White-crowned Sparrow (*Zonotrichia leucophrys*; G)	47	2	13	H_SISKIN1 (2), H_TUMIG07 (2), CATUST16 (1), H_PHYBOR04 (1), H_TUMIG06 (1), H_TABIO2 (1), H_MELCAR01 (2), H_TURDUS2 (1), H_VIGIL01 (1), H_DENEDE01 (1), P_MELMEL02 (1), P_GEOTRI09 (1)
Wilson's Warbler (*Cardellina pusilla*; G)	51	5	15	H_TURDUS2 (2), H_PHYBOR04 (2), H_SISKIN1 (1), H_MELCAR01 (2), H_TUMIG07 (1), H_VIGIL07 (2), H_GRBRU02 (1), H_VIGIL01 (1), H_DENADE01 (2), H_LAIRI01 (1), P_CATUST06 (2), P_GEOTRI09 (1), P_GASAN01 (2)
Yellow-rumped warbler (*Setophaga coronate*; T)	11	2	4	H_TABI10 (2), H_VIGIL07 (2), P_BAEBICO2 (2)

Letters presented in parentheses after each bird species indicate the nesting strategy used by that species. The letter “T” indicates tree nesters, “C” indicates cavity nesters, and “G” indicates ground nesters

### Haemosporidian parasite diversity

In total, we detected 10 *Plasmodium* and 29 *Haemoproteus* cytochrome b haplotypes. Thirty-three haplotypes had a 100% match to current sequences deposited in the MalAvi database (Table 2) [13], and the other six sequences were considered novel haplotypes (GenBank accession numbers MW147676, MW147677, MW147678, MW147679, MW147680, MW147681). The most common *Haemoproteus* lineages were TURDUS2 and SISKIN1, which were detected in 15 and 13 individuals, respectively. The most common *Plasmodium* lineage was PADOM11, which was detected in six individuals. Only one Warbling Vireo (*Vireo gilvus*) and two Lincoln’s Sparrows (*Melospiza lincolnii*) were found to be infected with both *Plasmodium* and *Haemoproteus*. The greatest parasite diversity was obtained from the Wilson’s Warbler (*Cardellina pusilla*; 13), the Warbling Vireo (12), and the White-crowned Sparrow (*Zonotrichia leucophrys*; 11), which also had some of the largest sample sizes.

### Haemoproteus

**Fig. 1:**
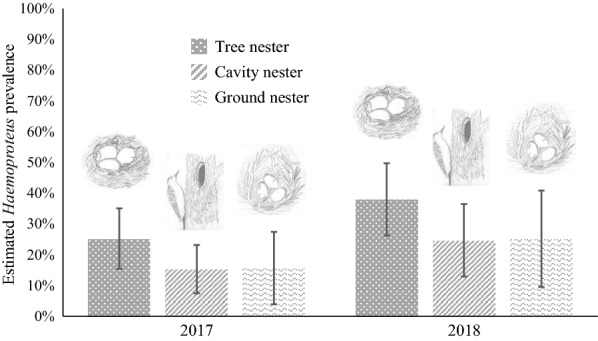
Estimated avian *Haemoproteus* prevalence (95% CI) for nest types and years in the Rocky Mountains

**Fig. 2:**
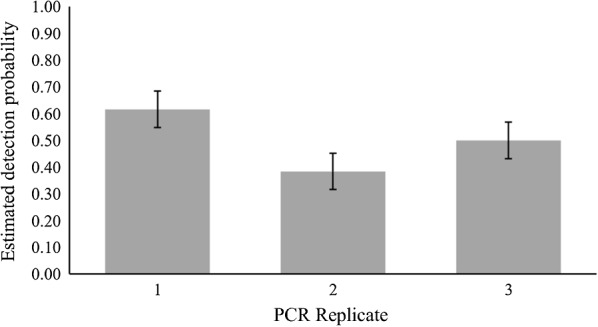
*Haemoproteus* detection probability (*p* ± SE) for PCR replicates in all-species analysis of birds in the Rocky Mountains

We detected *Haemoproteus* parasites in 109 out of 437 birds, a naïve (without taking detection probability or covariates into account) *Haemoproteus* prevalence of nearly 25% (109/437). Nest type and year were considered important variables associated with *Haemoproteus* prevalence in the all-species analysis, with variable weights of 0.54 and 0.85 (Table 3). Open-cup nesters in 2018 had the highest *Haemoproteus* overall prevalence, estimated at 38%, 95% CI 26.24%–49.76% (Fig. 1). Prevalence was similar for cavity and ground nesters in both years and was higher in 2018. In the all-species analysis, we found no evidence of unmodeled heterogeneity using a random effects model (Additional file 1: Table S1). PCR replicate was an important variable when considering detection probability, with a variable weight of 0.99. Detection probability was estimated at 0.62 (±0.07 SE) for the first PCR run, 0.38 (±0.07 SE) for the second PCR run, and 0.50 (±0.07 SE) for the third PCR run (Fig. 2).

**Table 3:** Cumulative Akaike information criterion (AICc) variable weights for variables considered in the all-species analysis for *Haemoproteus* prevalence

Variable	Variable weight
ψ(sex)	0.21
ψ(age)	0.05
ψ(migration)	0.19
*ψ(nest)*	*0.54*
*ψ(year)*	*0.85*
ψ(BCI)	0.27
*p(PCR run)*	*0.99*

Variables with a cumulative weight greater than 0.5 (italics) are considered important. ψ variables are those considered when evaluating prevalence. The *p* variables are those considered when evaluating detection probability. “PCR run”indicates the three PCR replicates carried out for each sample. “Nest” indicates nest type (open, ground, or cavity). BCI is body condition index defined as the ratio of body mass (g) to tarsus length (mm) for each individual

**Fig. 3:**
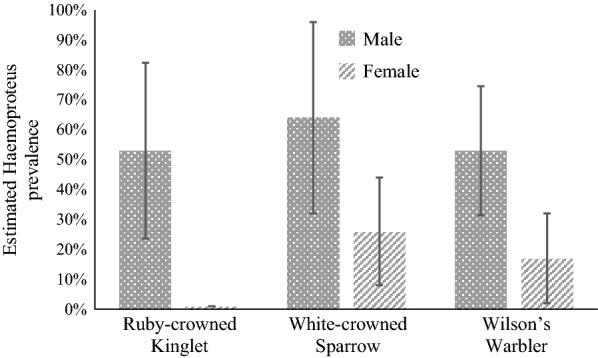
*Haemoproteus* prevalence (95% CI) for male and female Ruby-crowned Kinglet, White-crowned Sparrow, and Wilson’s Warbler

**Fig. 4:**
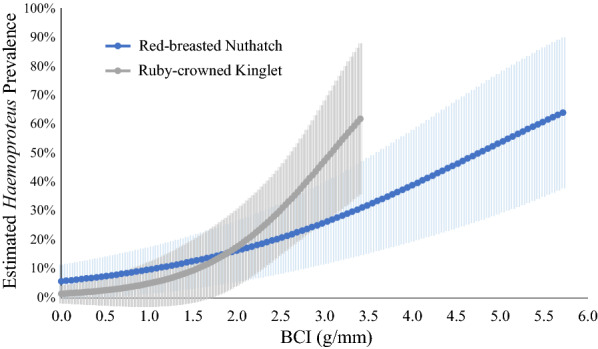
*Haemoproteus* prevalence (95% CI) and body condition index for Red-breasted Nuthatch and Ruby-crowned Kinglet

**Fig. 5:**
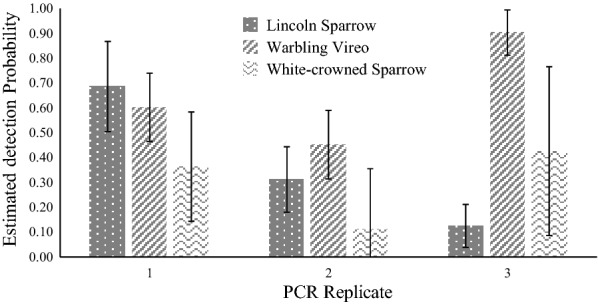
*Haemoproteus* detection probability (± SE) for PCR replicates in Lincoln Sparrow, Warbling Vireo, and White-crowned Sparrow

Among all species, the Warbling Vireo, American Robin (*Turdus migratorius*), and Wilson’s Warbler had the highest naïve *Haemoproteus* prevalence, at 59%, 35%, and 32%, respectively. In the species-specific analyses (Additional file 2: Table S2; Additional file 3: Table S3; Additional file 4: Table S4; Additional file 5: Table S5; Additional file 6: Table S6; Additional file 7: Table S7; Additional file 8: Table S8; Additional file 9: Table S9; Additional file 10: Table S10; Additional file 11: Table S11), all species had an estimated individual heterogeneity of nearly zero. In terms of prevalence (ψ), sex was considered an important variable for the Ruby-crowned Kinglet (*Regulus calendula*), the White-crowned Sparrow, and Wilson’s Warbler (Fig. 3), with variable weights of 0.57, 0.54, and 0.51, respectively (Table 4). Body condition index (BCI) was also considered an important covariate of prevalence for the Red-breasted Nuthatch (*Sitta canadensis*) and the Ruby-crowned Kinglet (Fig. 4), with variable weights of 0.69 and 0.84, respectively (Table 4). No species had variable weights above 0.5 for age or year. For detection probability (*p*), PCR run was an important variable for the Lincoln’s Sparrow and the White-crowned Sparrow, with variable weights of 0.96 and 0.50, respectively (Fig. 5).

**Table 4:** Cumulative variable Akaike information criterion (AICc) weights for variables considered in each species-specific analysis for *Haemoproteus* prevalence

Species	Cumulative Variable Weights				
	ψ(sex)	ψ(age)	ψ(year)	ψ(BCI)	p(PCR replicate)
American Robin	0.33	0.09	0.26	0.32	0.14
Lincoln Sparrow	0.29	0.25	0.27	0.26	*0.96*
Mountain Chickadee	–	–	0.22	0.23	0.06
Pine Siskin	0.27	0.25	0.34	0.39	0.24
Red-breasted Nuthatch	0.07	–	–	*0.69*	–
Ruby-crowned Kinglet	*0.57*	–	–	*0.84*	–
Dark-eyed Junco	0.35	0.05	0.03	0.21	0.30
Warbling Vireo	0.33	0.04	–	0.38	0.30
White-crowned Sparrow	*0.54*	0.10	0.40	0.35	*0.50*
Wilson's Warbler	*0.51*	0.07	0.42	0.25	0.08

Variables with a cumulative variable AICc weight greater than 0.5 (italics) are considered important. Cells with a dash indicate that models would not converge with the inclusion of that variable. ψ variables are those considered when evaluating prevalence. *p* variables are those considered when evaluating detection probability. “PCR run” indicates the three PCR replicates carried out for each sample. “Nest” indicates nest type (open, ground, or cavity). BCI is body condition index defined as the ratio of body mass (g) to tarsus length (mm) for each individual

### Plasmodium

We detected *Plasmodium* parasites in at least one PCR replicate in 23 out of 437 birds, which is a total naïve *Plasmodium* prevalence of 5.3%. When analyzing all species together, we detected no heterogeneity using a random effects model. We found no predictor variable with a cumulative variable AICc weight of at least 0.5, and therefore none of our hypothesized variables were considered important in predicting *Plasmodium* infection in the all-species analysis.

Wilson’s Warbler and Lincoln’s Sparrow had the greatest number of infected individuals per species, with three birds positive for *Plasmodium* in each. Because of the low number of positives per species, species-specific analyses could not be carried out for *Plasmodium* prevalence.

## Discussion

In this study, we present baseline knowledge of haemosporidian parasite presence, prevalence, and diversity across a suite of avian species in the Colorado Rocky Mountains. Among the 438 birds of 24 species sampled, 39 unique haemosporidian parasite haplotypes were detected, 21 host species had at least one infected individual, and *Haemoproteus* parasites had a larger host breadth and occurred at much higher prevalence than *Plasmodium*. In addition, six novel haplotypes were detected among three different species. Using an occupancy-modeling framework to account for imperfect detection of avian blood parasites, we found that nest type is an important species-level factor influencing *Haemoproteus* parasitism at our study site, with open-cup nesters having a higher prevalence than cavity and ground nesters. We also found that sex and BCI are important individual-level factors associated with *Haemoproteus* parasitism in some species, with males and birds with higher BCI having a higher blood parasite prevalence. In addition, we found that out of the 24 hatch-year birds that were sampled during the study, seven were positive for *Haemoproteus* blood parasites, meaning that local transmission was taking place at our sample site.

### Haemosporidian lineage diversity

Diversity of haemosporidian parasites in wild birds was high, with a total of 39 lineages of *Haemoproteus* and *Plasmodium* from 21 of the 24 avian species that were sampled (Table 2). *Plasmodium* parasites are considered generalists in terms of host breadth, while *Haemoproteus* are generally considered more host-specific [53]. However, we identified *Haemoproteus* in a wider range of bird species than *Plasmodium* and found that *Haemoproteus* parasites were more prevalent overall.

Despite the small sample sizes for some host species, some patterns were still apparent largely in the family Turdidae. One lineage, TURDUS2, infects many families of birds throughout Europe [60], Asia [61], and the United States [62], yet most detections have occurred in the Turdidae, including robins and other thrushes. Accordingly, American Robins at our study site had the highest proportion of TURDUS2 detections among species and may act as a reservoir for this parasite lineage. The lineage TUMIG07 has only been detected in the American Robin and Hermit Thrush (*Catharus guttatus*) in Alaska [62]. In our study, TUMIG07 was present in these species and six other hosts at our site. The VIGIL07 lineage has only been detected in the Vireonidae family in California [63], New Mexico (Marroquin-Flores unpublished data), and Michigan [42], and in our study the Warbling Vireo had the highest proportion of positive individuals. Of the seven detections of the TUMIG08 lineage in the MalAvi database, four have been from the American Robin [62]. Accordingly, two of the four detections of this lineage at our study site were from American Robins, with one detection in a Lincoln Sparrow and the other in a Warbling Vireo. The POETR01 lineage has been mainly detected in the Thrush family as well [61], and our one detection of this lineage was in an American Robin, in agreement with previous detections.

### Patterns across host species

When analyzing *Haemoproteus* prevalence across host species, nest type and year were important variables associated with infection (Fig. 1). Although some studies have found contradicting results [37, 38 and 64], open-cup nesting has been linked to higher *Haemoproteus* prevalence in many studies, such as Gonzales et al. [40], Smith et al. [42], and Fechio [43]. Higher prevalence in open-cup nesters may indicate that *Haemoproteus* vectors—biting midges—are more likely to come in contact with species that have open-cup nests than with ground or cavity nesters, perhaps because open-cup nesters are more vulnerable to exposure to blood feeding by the vectors.

Overall *Haemoproteus* prevalence also displayed marked variation between years in our study, with 2018 higher than 2017 (Fig. 3). Interannual variation in avian blood parasite prevalence is common and has been found in many studies, including Bensch et al. [64], Wood et al. [23], Lachish et al. [24], and Podmokła et al. [65]. One potential explanation is that the vectors responsible for *Haemoproteus* transmission fluctuate in abundance in response to weather variation (e.g., temperature and rainfall), which alters the habitat and microclimate they require for breeding to conditions that are more favorable for vectors. Alternatively, annual variation in host demography and population dynamics could also play a role in driving this annual variation in prevalence [66, 67].

Age, sex, BCI, and migration were not considered important variables across species in our study, although they have been linked to higher haemosporidian parasite prevalence in other studies, e.g., Hatchwell et al. [68], Deviche et al. [69], Garvin et al. [70], and Calero-Riestra and Garcia [71]. Further studies are needed to address the influence of host traits on patterns of avian haemosporidian parasite infection and to determine whether such patterns exist and persist at larger spatial scales and across a wider host–parasite community.

When analyzing *Plasmodium* prevalence among host species, no associations were found between prevalence and species-level traits, likely due to the low number of individuals that were positive for the parasite. Elevation governs the distribution of parasites belonging to different genera, with *Plasmodium* parasites being more prevalent at lower altitudes and *Haemoproteus* parasite prevalence increasing with elevation [25]; our site is very high, which may explain the lack of *Plasmodium* detections. Accordingly, Eisen et al. [36] found that *Culex* spp. mosquitoes, the main vectors of *Plasmodium* parasites, were not yet established in areas in and around Rocky Mountain National Park at similar elevations as our study site. Associations between exposure to mosquitoes and *Plasmodium* prevalence across host species has been demonstrated [28], supporting the idea that *Plasmodium* vectors may be absent or present in low numbers at our study site.

### Patterns of *Haemoproteus* infection within individual species

Sex was associated with *Haemoproteus* infection in the Ruby-crowned Kinglet, White-crowned Sparrow, and Wilson’s Warbler (Fig. 4). Sex-related differences in haemosporidian parasite prevalence are often observed in nature; however, sex bias in parasitism often varies between and within host–parasite systems [72]. Contrary to our prediction, our study demonstrated a strong male-biased parasite prevalence in the three species mentioned above, with the Ruby-crowned Kinglet having the largest difference between sexes (53% in males vs. 1% in females). Although the greater stress of reproduction in females might translate to weakened immune responses and higher prevalence [73], there is overwhelming evidence that sex-associated hormones can directly influence the differential susceptibility of each sex to infections [74]. For example, testosterone has immunosuppressive effects in many species, leading to higher susceptibility of males to parasite infections [75]. This is not the case for every host–parasite relationship, as was illustrated by the lack of an association between parasite prevalence and sex in the other seven species that we analyzed.

Body condition index (BCI) was positively associated with *Haemoproteus* infection in the Red-breasted Nuthatch and the Ruby-crowned Kinglet when analyzed separately (Fig. 5). Similar results have been found in the American Kestrel (*Falco sparverius*), the Yellow-rumped Warbler (*Setophaga coronate*), and the Great Tit (*Parus major*) [78, 79]. Although the reason for this positive correlation is unknown, it may be due to the lower capture probability in infected individuals. If infected individuals with low body condition are less active and are less likely to fly into mist nets, that leaves only infected individuals with greater body condition to be caught. Similarly, if individuals with low body condition are unable to survive the acute stage of *Haemoproteus* infection, then this may leave more infected individuals with higher body condition. The other eight species in our study did not show an apparent relationship between prevalence and BCI, which is a common result in wildlife studies given that host condition and its responsiveness to infection could change in response to foraging resources that fluctuate in space and time [80, 81]. Some parasites cause minimal or no effects on condition in certain host taxa [81], and some infections might exert negative fitness effects only during stressful periods or under resource limitation [82, 83].

We found no relationship between *Haemoproteus* prevalence and individual-level traits (sex, age, BCI) in the American Robin, Mountain Chickadee (*Poecile gambeli*), Pine Siskin (*Carduelis pinus*), or Dark-eyed Junco (*Junco hyemalis*), contrary to our hypotheses. Our results suggest that the individual-level traits examined in this study may not be important predictors of *Haemoproteus* infection for all species.

### Detection probability

PCR replicate was an important variable associated with detection probability for *Haemoproteus* infection for three species (Lincoln Sparrow, Warbling Vireo, and White-Crowned Sparrow) as well as for the all-species analysis, with PCR results varying among the three PCR runs (Figs. 2 and 5). Nested PCR assays for haemosporidian parasites are known to be vulnerable to false-negative results for samples with low parasite intensities [84], which is most likely responsible for the variation we found between PCR replicates.

Detection probability for *Plasmodium* parasites was lower than for *Haemoproteus*, consistent with the idea of lower detection in blood samples [84], because *Plasmodium* enters latent, exoerythrocytic phases during chronic infection and may even be absent in the blood stream [10]. Thus, sampling peripheral blood may not allow for detection of all true infections with *Plasmodium*, leading to underestimates of prevalence.

## Conclusion

Our results suggest that open-cup nesting birds in the Colorado Rocky Mountains are commonly infected with avian blood parasites, and that male Ruby-crowned Kinglets, White-crowned Sparrows, and Wilson’s Warblers have higher prevalence than females of these species. We present baseline knowledge of blood parasite presence, prevalence, and diversity across avian species in the Colorado Rocky Mountains, providing a strong foundation for subsequent studies. With climate change expected to support the expansion of avian blood parasite distributions, such that monitoring avian haemosporidian parasites should continue in order to detect potential climate-related changes in prevalence and diversity over time. Our study is the only avian blood parasite survey conducted in the Colorado Rocky Mountains, to date; additional research in this area examining host–parasite relationships would help to determine whether anthropogenic changes–such as climate change–leading to potential changes in vector communities or parasite distributions may pose a threat to resident avian populations.

## Supplementary Information

**Additional file 1: Table S1. ** Model rankings exploring factors affecting detection probability (p) and prevalence (ψ) of *Haemoproteus* parasites across host species.

**Additional file 2: Table S2. ** Model rankings exploring factors affecting detection probability (p) and prevalence (ψ) of *Haemoproteus* parasites in American Robins.

**Additional file 3: Table S3.** Model rankings exploring factors affecting detection probability (p) and prevalence (ψ) of *Haemoproteus* parasites in Lincoln’s Sparrows.

**Additional file 4: Table S4.** Model rankings exploring factors affecting detection probability (p) and prevalence (ψ) of *Haemoproteus* parasites in Mountain Chickadees.

**Additional file 5: Table S5.** Model rankings exploring factors affecting detection probability (p) and prevalence (ψ) of *Haemoproteus* parasites in Pine Siskins.

** Additional file 6: Table S6. ** Model rankings exploring factors affecting detection probability (p) and prevalence (ψ) of *Haemoproteus* parasites in Red-breasted Nuthatches.

**Additional file 7: Table S7.** Model rankings exploring factors affecting detection probability (p) and prevalence (ψ) of *Haemoproteus* parasites in Ruby-crowned Kinglets.

**Additional file 8: Table S8. ** Model rankings exploring factors affecting detection probability (p) and prevalence (ψ) of *Haemoproteus* parasites in Dark-eyed Juncos.

**Additional file 9: Table S9.** Model rankings exploring factors affecting detection probability (p) and prevalence (ψ) of *Haemoproteus* parasites in Warbling Vireos.

**Additional file 10: Table S10.** Model rankings exploring factors affecting detection probability (p) and prevalence (ψ) of *Haemoproteus* parasites in White-crowned Sparrows.

**Additional file 11: Table S11.** Model rankings exploring factors affecting detection probability (p) and prevalence (ψ) of *Haemoproteus* parasites in Wilson’s Warblers.

## Data Availability

The data sets used and/or analyzed during the current study are available from the corresponding author on reasonable request.
