# Supplementary material for: Sex and nest type influence avian blood parasite prevalence in a high-elevation bird community
Source: Parasit Vectors. 2021 Mar 8;14:145. doi: 10.1186/s13071-021-04612-w (PMC7938522; doi:10.1186/s13071-021-04612-w)
Supplement: Supplementary file 1 — Additional file 1: Table S1. Model rankings exploring factors affecting detection probability (p) and prevalence (ψ) of Haemoproteus parasites across host species. [file 13071_2021_4612_MOESM1_ESM.pdf]

Additional File 1 Model rankings exploring factors affecting detection probability (p) and prevalence (ψ) of *Haemoproteus* parasites across host species.

Model	K	ΔAICc	w_i	Deviance
σ(.) + p(PCR run) + ψ (nest+year)	7	0.00	0.30	891.71
σ(.) + p(PCR run) + ψ (year)	6	1.58	0.13	895.35
σ(.) + p(PCR run) + ψ (year+migration)	7	3.41	0.05	895.12
σ(.) + p(PCR run) + ψ (sex+year)	7	3.63	0.05	895.34
σ(.) + p(PCR run) + ψ (year+BCI)	7	3.64	0.05	895.35
σ(.) + p(PCR run) + ψ (sex+nest+year)	9	4.16	0.04	891.70
σ(.) + p(PCR run) + ψ (nest+year+BCI)	9	4.16	0.04	891.71
σ(.) + p(PCR run) + ψ (nest+year+migration)	9	4.16	0.04	891.71
σ(.) + p(PCR run) + ψ(BCI)	6	5.07	0.02	898.84
σ(.) + p(PCR run) + ψ(nest+BCI)	8	5.30	0.02	894.94
σ(.) + p(PCR run) + ψ(sex+year+migration)	8	5.49	0.02	895.12
σ(.) + p(PCR run) + ψ(year+BCI+migration)	8	5.49	0.02	895.12
σ(.) + p(PCR run) + ψ(sex+year+BCI)	8	5.70	0.02	895.33
σ(.) + p(PCR run) + ψ(.)	5	6.15	0.01	901.98
σ(.) + p(PCR run) + ψ(sex+nest+year+BCI)	10	6.25	0.01	891.70
σ(.) + p(PCR run) + ψ(sex+nest+year+migration)	10	6.25	0.01	891.70
σ(.) + p(PCR run) + ψ(nest+year+BCI+migration)	10	6.25	0.01	891.71
σ(.) + p(PCR run) + ψ(BCI+migration)	7	6.30	0.01	898.01
σ(.) + p(PCR run) + ψ(nest)	7	6.34	0.01	898.05
σ(.) + p(PCR run) + ψ(sex+BCI)	7	7.01	0.01	898.72
σ(.) + p(PCR run) + ψ(age+year)	9	7.04	0.01	894.59
σ(.) + p(PCR run) + ψ(nest+BCI+migration)	9	7.18	0.01	894.73
σ(.) + p(PCR run) + ψ(sex+nest+BCI)	9	7.36	0.01	894.91

σ(.) + p(PCR run) + ψ(age+nest+year)	11	7.43	0.01	890.78
σ(.) + p(PCR run) + ψ(sex+year+BCI+migration)	9	7.57	0.01	895.12
σ(.) + p(PCR run) + ψ(migration)	6	7.64	0.01	901.42
σ(.) + p(PCR run) + ψ(sex)	6	8.03	0.01	901.80
σ(.) + p(PCR run) + ψ(sex+BCI+migration)	8	8.33	0.00	897.97
σ(.) + p(PCR run) + ψ(nest+migration)	8	8.35	0.00	897.98
σ(.) + p(PCR run) + ψ(sex+nest)	8	8.35	0.00	897.98
σ(.) + p(PCR run) + ψ(sex+nest+year+BCI+migration)	11	8.35	0.00	891.70
σ(.) + p(.) + ψ(year)	3	8.45	0.00	908.37
σ(.) + p(PCR run) + ψ(age+year+migration)	10	8.95	0.00	894.40
σ(.) + p(PCR run) + ψ(sex+age+year)	10	9.13	0.00	894.58
σ(.) + p(PCR run) + ψ(age+year+BCI)	10	9.13	0.00	894.58
σ(.) + p(PCR run) + ψ(sex+nest+BCI+migration)	10	9.26	0.00	894.71
σ(.) + p(PCR run) + ψ(sex+age+nest+year)	12	9.54	0.00	890.78
σ(.) + p(PCR run) + ψ(age+nest+year+migration)	12	9.54	0.00	890.78
σ(.) + p(PCR run) + ψ(age+nest+year+BCI)	12	9.55	0.00	890.78
σ(.) + p(PCR run) + ψ(sex+migration)	7	9.61	0.00	901.32
σ(.) + p(.) + ψ(year+migration)	4	10.26	0.00	908.14
σ(.) + p(PCR run) + ψ(sex+nest+migration)	9	10.38	0.00	897.93
σ(.) + p(.) + ψ(sex+year)	4	10.47	0.00	908.35
σ(.) + p(PCR run) + ψ(age+BCI)	9	10.78	0.00	898.33
σ(.) + p(.) + ψ(nest+year)	6	10.95	0.00	904.72
σ(.) + p(PCR run) + ψ(age+nest+BCI)	11	11.01	0.00	894.35
σ(.) + p(PCR run) + ψ(sex+age+year+migration)	11	11.05	0.00	894.40
σ(.) + p(PCR run) + ψ(age+year+BCI+migration)	11	11.05	0.00	894.40
σ(.) + p(PCR run) + ψ(sex+age+year+BCI)	11	11.22	0.00	894.57
σ(.) + p(PCR run) + ψ(sex+age+nest+year+migration)	13	11.67	0.00	890.78
σ(.) + p(PCR run) + ψ(sex+age+nest+year+BCI)	13	11.67	0.00	890.78
σ(.) + p(PCR run) + ψ(age+nest+year+BCI+migration)	13	11.67	0.00	890.78

σ(.) + p(PCR run) + ψ(age)	8	11.75	0.00	901.38
σ(.) + p(PCR run) + ψ(age+nest)	10	12.00	0.00	897.46
σ(.) + p(PCR run) + ψ(age+BCI+migration)	10	12.17	0.00	897.63
σ(.) + p(.) + ψ(year+BCI)	5	12.53	0.00	908.36
σ(.) + p(PCR run) + ψ(sex+age+BCI)	10	12.76	0.00	898.21
σ(.) + p(PCR run) + ψ(age+nest+BCI+migration)	12	12.97	0.00	894.20
σ(.) + p(.) + ψ(sex+nest+year)	7	13.01	0.00	904.72
σ(.) + p(.) + ψ(nest+year+BCI)	7	13.01	0.00	904.72
σ(.) + p(.) + ψ(nest+year+migration)	7	13.01	0.00	904.72
σ(.) + p(PCR run) + ψ(sex+age+nest+BCI)	12	13.08	0.00	894.32
σ(.) + p(PCR run) + ψ(sex+age+year+BCI+migration)	12	13.16	0.00	894.40
σ(.) + p(PCR run) + ψ(age+migration)	9	13.43	0.00	900.98
σ(.) + p(PCR run) + ψ(sex+age)	9	13.65	0.00	901.20
σ(.) + p(PCR run) + ψ(sex+age+nest+year+BCI+migration)	14	13.80	0.00	890.77
σ(.) + p(.) + ψ(BCI)	4	13.98	0.00	911.86
σ(.) + p(PCR run) + ψ(sex+age+nest)	11	14.03	0.00	897.38
σ(.) + p(PCR run) + ψ(age+nest+migration)	11	14.08	0.00	897.43
σ(.) + p(.) + ψ(nest+BCI)	6	14.17	0.00	907.95
σ(.) + p(PCR run) + ψ(sex+age+BCI+migration)	11	14.23	0.00	897.58
σ(.) + p(.) + ψ(sex+year+migration)	6	14.36	0.00	908.13
σ(.) + p(.) + ψ(year+BCI+migration)	6	14.36	0.00	908.13
σ(.) + p(.) + ψ(sex+year+BCI)	6	14.57	0.00	908.35
σ(.) + p(.) + ψ(.)	3	15.08	0.00	914.99
σ(.) + p(PCR run) + ψ(sex+age+nest+BCI+migration)	13	15.08	0.00	894.19
σ(.) + p(.) +ψ(sex+nest+year+BCI)	8	15.08	0.00	904.72
σ(.) + p(.) + ψ(sex+nest+year+migration)	8	15.08	0.00	904.72
σ(.) + p(.) + ψ(nest+year+BCI+migration)	8	15.09	0.00	904.72
σ(.) + p(.) + ψ(BCI+migration)	5	15.19	0.00	911.02
σ(.) + p(.) + ψ(nest)	5	15.24	0.00	911.07

σ(.) + p(PCR run) + ψ(sex+age+migration)	10	15.42	0.00	900.87
σ(.) + p(.) + ψ(age+year)	7	15.90	0.00	907.60
σ(.) + p(.) +ψ(sex+BCI)	5	15.91	0.00	911.74
σ(.) + p(.) + ψ(nest+BCI+migration)	7	16.03	0.00	907.74
σ(.) + p(PCR run) + ψ(sex+age+nest+migration)	12	16.13	0.00	897.37
σ(.) + p(.) + ψ(sex+nest+BCI)	7	16.21	0.00	907.92
σ(.) + p(.) +ψ(age+nest+year)	9	16.25	0.00	903.80
σ(.) + p(.) +ψ(sex+year+BCI+migration)	7	16.42	0.00	908.13
σ(.) + p(.) + ψ(migration)	4	16.55	0.00	914.43
σ(.) + p(.) +ψ(sex)	4	16.94	0.00	914.82
σ(.) + p(.) +ψ(sex+nest+year+BCI+migration)	9	17.17	0.00	904.72
σ(.) + p(.) + ψ(sex+BCI+migration)	6	17.21	0.00	910.98
σ(.) + p(.) + ψ(nest+migration)	6	17.22	0.00	910.99
σ(.) + p(.) + ψ(sex+nest)	6	17.22	0.00	911.00
σ(.) + p(.) + ψ(age+year+migration)	8	17.78	0.00	907.41
σ(.) + p(.) + ψ(sex+age+year)	8	17.96	0.00	907.59
σ(.) + p(.) + ψ(age+year+BCI)	8	17.96	0.00	907.60
σ(.) + p(.) + ψ(sex+nest+BCI+migration)	8	18.09	0.00	907.73
σ(.) + p(.) + ψ(sex+age+nest+year)	10	18.34	0.00	903.79
σ(.) + p(.) + ψ(age+nest+year+migration)	10	18.34	0.00	903.79
σ(.) + p(.) + ψ(age+nest+year+BCI)	10	18.34	0.00	903.79
σ(.) + p(.) + ψ(sex+migration)	5	18.51	0.00	914.34
σ(.) + p(.) + ψ(sex+nest+migration)	7	19.23	0.00	910.94
σ(.) + p(.) + ψ(age+BCI)	7	19.64	0.00	911.35
σ(.) + p(.) + ψ(age+nest+BCI)	9	19.82	0.00	907.37
σ(.) + p(.) + ψ(sex+age+year+migration)	9	19.86	0.00	907.41
σ(.) + p(.) + ψ(age+year+BCI+migration)	9	19.87	0.00	907.41
σ(.) + p(.) + ψ(sex+age+year+BCI)	9	20.04	0.00	907.59
σ(.) + p(.) + ψ(sex+age+nest+year+migration)	11	20.44	0.00	903.79

σ(.) + p(.) + ψ(sex+age+nest+year+BCI)	11	20.44	0.00	903.79
σ(.) + p(.) + ψ(age+nest+year+BCI+migration)	11	20.44	0.00	903.79
σ(.) + p(.) + ψ(age)	6	20.62	0.00	914.40
σ(.) + p(.) + ψ(age+nest)	8	20.84	0.00	910.47
σ(.) + p(.) + ψ(age+BCI+migration)	8	21.01	0.00	910.64
σ(.) + p(.) + ψ(sex+age+BCI)	8	21.59	0.00	911.22
σ(.) + p(.) + ψ(age+nest+BCI+migration)	10	21.76	0.00	907.22
σ(.) + p(.) + ψ(sex+age+nest+BCI)	10	21.88	0.00	907.33
σ(.) + p(.) + ψ(sex+age+year+BCI+migration)	10	21.96	0.00	907.41
σ(.) + p(.) + ψ(age+migration)	7	22.28	0.00	913.99
σ(.) + p(.) + ψ(sex+age)	7	22.51	0.00	914.22
σ(.) + p(.) + ψ(sex+age+nest+year+BCI+migration)	12	22.55	0.00	903.79
σ(.) + p(.) + ψ(sex+age+nest)	9	22.85	0.00	910.40
σ(.) + p(.) + ψ(age+nest+migration)	9	22.90	0.00	910.45
σ(.) + p(.) + ψ(sex+age+BCI+migration)	9	23.04	0.00	910.59
σ(.) + p(.) + ψ(sex+age+nest+BCI+migration)	11	23.85	0.00	907.20
σ(.) + p(.) + ψ(sex+age+migration)	8	24.25	0.00	913.88
σ(.) + p(.) + ψ(sex+age+nest+migration)	10	24.93	0.00	910.38

Model rankings of the complete set of models exploring factors affecting detection probability (p) and prevalence (ψ) of *Haemoproteus* parasites across host species. Host bird species were captured and sampled at a high-elevation valley in northern Colorado during 2017-2018. 'PCR run' indicates the 3 PCR replicates carried out for each sample. 'Nest' indicates nest type (open, ground, or cavity). BCI is body condition index defined as the ration of body mass (g) to tarsus length (mm) for each individual. The number of parameters (K), model weights (w_i), and deviance are shown for each model and the

models are ranked by their AICc differences relative to the best model in the set ($\Delta AICc_i$). Sigma (σ) was a random effect included in every model to account for unmodeled heterogeneity.
