# Supplementary material for: Sex and nest type influence avian blood parasite prevalence in a high-elevation bird community
Source: Parasit Vectors. 2021 Mar 8;14:145. doi: 10.1186/s13071-021-04612-w (PMC7938522; doi:10.1186/s13071-021-04612-w)
Supplement: Supplementary file 2 — Additional file 2: Table S2. Model rankings exploring factors affecting detection probability (p) and prevalence (ψ) of Haemoproteus parasites in American Robins. [file 13071_2021_4612_MOESM2_ESM.pdf]

Additional File 2 Model rankings exploring factors affecting detection probability (p) and prevalence (ψ) of *Haemoproteus* parasites in American Robins.

Model	K	ΔAICc	w_i	Deviance
σ(.) + p(.) + ψ(.)	3	0.00	0.33	104.05
σ(.) + p(.) + ψ(sex)	4	1.66	0.15	103.23
σ(.) + p(.) + ψ(BCI)	4	1.66	0.15	103.24
σ(.) + p(.) + ψ(year)	4	2.44	0.10	104.01
σ(.) + p(.) + ψ(sex+BCI)	5	3.28	0.06	102.23
σ(.) + p(.) + ψ(year+BCI)	5	3.34	0.06	102.29
σ(.) + p(.) + ψ(sex+year)	5	4.27	0.04	103.22
σ(.) +p(PCR run) + ψ(.)	5	4.90	0.03	103.85
σ(.) + p(.) + ψ(sex+year+BCI)	6	5.61	0.02	101.79
σ(.) + p(PCR run) + ψ(sex)	6	6.86	0.01	103.04
σ(.) + p(run) + ψ(BCI)	6	6.87	0.01	103.04
σ(.) + p(.) + ψ(age)	6	6.90	0.01	103.07
σ(.) + p(PCR run) + ψ(year)	6	7.64	0.01	103.81
σ(.) + p(PCR run) + ψ(sex+BCI)	7	8.81	0.00	102.03
σ(.) + p(PCR run) + ψ(year+BCI)	7	8.88	0.00	102.10
σ(.) + p(.) + ψ(sex+age)	7	9.35	0.00	102.57
σ(.) + p(.) + ψ(age+BCI)	7	9.41	0.00	102.63
σ(.) + p(.) + ψ(age+year)	7	9.78	0.00	103.00
σ(.) + p(PCR run) + ψ(sex+year)	7	9.81	0.00	103.02
σ(.) + p(PCR run) + ψ(sex+year+BCI)	8	11.52	0.00	101.59
σ(.) + p(.) + ψ(age+year+BCI)	8	11.67	0.00	101.74
σ(.) + p(.) + ψ(sex+age+BCI)	8	11.86	0.00	101.94
σ(.) + p(.) + ψ(sex+age+year)	8	12.50	0.00	102.57
σ(.) + p(PCR run) + ψ(age)	8	12.80	0.00	102.88

σ(.) + p(.) + ψ(sex+age+year+BCI)	9	14.75	0.00	101.47
σ(.) + p(PCR run) + ψ(sex+age)	9	15.66	0.00	102.37
σ(.) + p(PCR run) + ψ(age+BCI)	9	15.72	0.00	102.43
σ(.) + p(PCR run) + ψ(age+year)	9	16.08	0.00	102.80
σ(.) + p(PCR run) + ψ(age+year+BCI)	10	18.41	0.00	101.54
σ(.) + p(PCR run) + ψ(sex+age+BCI)	10	18.61	0.00	101.74
σ(.) + p(PCR run) + ψ(sex+age+year)	10	19.24	0.00	102.37
σ(.) + p(PCR run) + ψ(sex+age+year+BCI)	11	21.99	0.00	101.27

Model set and rankings exploring the importance of factors affecting the detection probability (p) and prevalence (ψ) of *Haemoproteus* blood parasites in American Robins captured and sampled at a high-elevation valley in northern Colorado during 2017-2018. 'PCR run' indicates the 3 PCR replicates carried out for each sample. The number of parameters (K), model weights (w_i), and deviance are shown for each model and the models are ranked by their AICc differences relative to the best model in the set (Δ**AICc**$_i$). Sigma (σ) was a random effect included in every model to account for unmodeled heterogeneity.
