# Supplementary material for: Sex and nest type influence avian blood parasite prevalence in a high-elevation bird community
Source: Parasit Vectors. 2021 Mar 8;14:145. doi: 10.1186/s13071-021-04612-w (PMC7938522; doi:10.1186/s13071-021-04612-w)
Supplement: Supplementary file 3 — Additional file 3: Table S3. Model rankings exploring factors affecting detection probability (p) and prevalence (ψ) of Haemoproteus parasites in Lincoln’s Sparrows. [file 13071_2021_4612_MOESM3_ESM.pdf]

Additional File 3 Model rankings exploring factors affecting detection probability (p) and prevalence (ψ) of *Haemoproteus* parasites in Lincoln's Sparrows.

Model	K	ΔAICc	w_i	Deviance
σ(.) + p(PCR run) + ψ(.)	4	0.00	0.27	102.29
σ(.) + p(PCR run) + ψ(sex)	5	1.56	0.12	101.51
σ(.) + p(PCR run) + ψ(year)	5	2.04	0.10	101.99
σ(.) + p(PCR run) + ψ(BCI)	5	2.10	0.10	102.06
σ(.) + p(PCR run) + ψ(age)	7	2.29	0.09	97.33
σ(.) + p(PCR run)+ψ(age+year)	8	3.65	0.04	96.10
σ(.) + p(PCR run) + ψ(sex+year)	6	3.67	0.04	101.21
σ(.) + p(PCR run) + ψ(age+BCI)	8	3.68	0.04	96.14
σ(.) + p(PCR run) + ψ(sex+BCI)	6	3.72	0.04	101.26
σ(.) + p(PCR run) + ψ(year+BCI)	6	4.45	0.03	101.98
σ(.) + p(PCR run) + ψ(sex+age)	8	4.61	0.03	97.07
σ(.) + p(PCR run) + ψ(sex+age+year)	9	6.11	0.01	95.89
σ(.) + p(PCR run) + ψ(sex+age+BCI)	9	6.13	0.01	95.91
σ(.) + p(PCR run) + ψ(sex+year+BCI)	7	6.17	0.01	101.21
σ(.) + p(PCR run) + ψ(age+year+BCI)	9	6.31	0.01	96.09
σ(.) + p(.) + ψ(.)	2	6.57	0.01	113.32
σ(.) + p(.) + ψ(sex)	3	7.98	0.01	112.54
σ(.) + p(.) + ψ(age)	5	8.40	0.00	108.36
σ(.) + p(.) + ψ(year)	3	8.46	0.00	113.02
σ(.) + p(.) + ψ(BCI)	3	8.53	0.00	113.08
σ(.) + p(PCR run) + ψ(sex+age+year+BCI)	10	8.86	0.00	95.87
σ(.) + p(.) + ψ(age+year)	6	9.67	0.00	107.21
σ(.) + p(.) + ψ(age+BCI)	6	9.74	0.00	107.27
σ(.) + p(.) + ψ(sex+year)	4	9.94	0.00	112.24

σ(.) + p(.) + ψ(sex+BCI)	4	9.99	0.00	112.28
σ(.) + p(.) + ψ(sex+age)	6	10.62	0.00	108.16
σ(.) + p(.) + ψ(year+BCI)	4	10.72	0.00	113.01
σ(.) + p(.) + ψ(sex+age+year)	7	11.99	0.00	107.03
σ(.) + p(.) + ψ(sex+age+BCI)	7	12.05	0.00	107.09
σ(.) + p(.) + ψ(age+year+BCI)	7	12.16	0.00	107.21
σ(.) + p(.) + ψ(sex+year+BCI)	5	12.28	0.00	112.23
σ(.) + p(.) + ψ(sex+age+year+BCI)	8	14.58	0.00	107.03

Model set and rankings exploring the importance of factors affecting the detection probability (p) and prevalence (ψ) of *Haemoproteus* blood parasites in Lincoln Sparrows captured and sampled at a high-elevation valley in northern Colorado during 2017-2018. 'PCR run' indicates the 3 PCR replicates carried out for each sample. The number of parameters (K), model weights (w_i), and deviance are shown for each model and the models are ranked by their AICc differences relative to the best model in the set ($\Delta AICc_i$). Sigma (σ) was a random effect included in every model to account for unmodeled heterogeneity.
