# Supplementary material for: Sex and nest type influence avian blood parasite prevalence in a high-elevation bird community
Source: Parasit Vectors. 2021 Mar 8;14:145. doi: 10.1186/s13071-021-04612-w (PMC7938522; doi:10.1186/s13071-021-04612-w)
Supplement: Supplementary file 4 — Additional file 4: Table S4. Model rankings exploring factors affecting detection probability (p) and prevalence (ψ) of Haemoproteus parasites in Mountain Chickadees. [file 13071_2021_4612_MOESM4_ESM.pdf]

Additional File 4 Model rankings exploring factors affecting detection probability (p) and prevalence (ψ) of *Haemoproteus* parasites in Mountain Chickadees.

Model	K	ΔAICc	w_i	Deviance
σ(.) + p(.) + ψ(.)	2	0.00	0.55	35.57
σ(.) + p(.) + ψ(BCI)	3	2.23	0.18	35.01
σ(.) + p(.) + ψ(year)	3	2.30	0.17	35.08
σ(.) + p(.) + ψ(year + BCI)	4	5.24	0.04	34.85
σ(.) + p(PCR run) + ψ(.)	4	5.35	0.04	34.97
σ(.) + p(PCR run) + ψ(BCI)	5	8.40	0.01	34.40
σ(.) + p(PCR run) + ψ(year)	5	8.48	0.01	34.47
σ(.) + p(PCR run)+ψ(year+BCI)	6	12.43	0.00	34.24

Model set and rankings exploring the importance of factors affecting the detection probability (p) and prevalence (ψ) of *Haemoproteus* blood parasites in for Mountain Chickadees captured and sampled at a high-elevation valley in northern Colorado during 2017-2018. 'PCR run' indicates the 3 PCR replicates carried out for each sample. The number of parameters (K), model weights (w_i), and deviance are shown for each model and the models are ranked by their AICc differences relative to the best model in the set ($\Delta AICc_i$). Sigma (σ) was a random effect included in every model to account for unmodeled heterogeneity.
