# Supplementary material for: Sex and nest type influence avian blood parasite prevalence in a high-elevation bird community
Source: Parasit Vectors. 2021 Mar 8;14:145. doi: 10.1186/s13071-021-04612-w (PMC7938522; doi:10.1186/s13071-021-04612-w)
Supplement: Supplementary file 5 — Additional file 5: Table S5. Model rankings exploring factors affecting detection probability (p) and prevalence (ψ) of Haemoproteus parasites in Pine Siskins. [file 13071_2021_4612_MOESM5_ESM.pdf]

Additional File 5 Model rankings exploring factors affecting detection probability (p) and prevalence (ψ) of *Haemoproteus* parasites in Pine Siskins.

Model	K	ΔAICc	w_i	Deviance
σ(.) + p(.) + ψ(.)	2	0.00	0.14	57.14
σ(.) + p(.) + ψ(BCI)	3	0.29	0.12	55.09
σ(.) + p(.) + ψ(year)	3	0.57	0.11	55.37
σ(.) + p(.) + ψ(age)	3	1.76	0.06	56.56
σ(.) + p(.) + ψ(sex+BCI)	4	1.88	0.06	54.20
σ(.) + p(PCR run) + ψ(.)	4	1.98	0.05	54.30
σ(.) + p(.) + ψ(sex)	3	2.29	0.05	57.09
σ(.) + p(.) + ψ(sex+year)	4	2.36	0.04	54.69
σ(.) + p(PCR run) + ψ(BCI)	5	2.55	0.04	52.24
σ(.) + p(.) + ψ(age+BCI)	4	2.60	0.04	54.93
σ(.) + p(.) + ψ(BCI+year)	4	2.76	0.04	55.08
σ(.) + p(PCR run) + ψ(year)	5	2.83	0.03	52.53
σ(.) + p(.) + ψ(age+year)	4	2.83	0.03	55.16
σ(.) + p(PCR run) + ψ(age)	5	4.02	0.02	53.72
σ(.) + p(.) + ψ(sex+age)	4	4.08	0.02	56.41
σ(.) + p(.) + ψ(sex+age+BCI)	5	4.38	0.02	54.08
σ(.) + p(PCR run) + ψ(sex+BCI)	6	4.45	0.02	51.37
σ(.) + p(.) + ψ(sex+BCI+year)	5	4.50	0.01	54.20
σ(.) + p(PCR run) + ψ(sex)	5	4.55	0.01	54.25
σ(.) + p(.) + ψ(sex+age+year)	5	4.76	0.01	54.46
σ(.) + p(PCR run) + ψ(sex+year)	6	4.91	0.01	51.83
σ(.) + p(PCR run) + ψ(age+BCI)	6	5.17	0.01	52.08
σ(.) + p(.) + ψ(age+BCI+year)	5	5.23	0.01	54.92
σ(.) + p(PCR run) + ψ(BCI+year)	6	5.33	0.01	52.24

σ(.) + p(PCR run) + ψ(age+year)	6	5.39	0.01	52.31
σ(.) + p(PCR run) + ψ(sex+age)	6	6.64	0.01	53.56
σ(.) + p(.) + ψ(sex+age+BCI+year)	6	7.16	0.00	54.08
σ(.) + p(.) + ψ(sex+age+BCI)	7	7.28	0.00	51.24
σ(.) + p(PCR run) + ψ(sex+BCI+year)	7	7.41	0.00	51.37
σ(.) + p(PCR run) + ψ(sex+age+year)	7	7.63	0.00	51.60
σ(.) + p(PCR run) + ψ(age+BCI+year)	7	8.12	0.00	52.08
σ(.) + p(PCR run) + ψ(sex+age+BCI+year)	8	10.42	0.00	51.24

Model set and rankings exploring the importance of factors affecting the detection probability (p) and prevalence (ψ) of *Haemoproteus* blood parasites in for Pine Siskins captured and sampled at a high-elevation valley in northern Colorado during 2017-2018. 'PCR run' indicates the 3 PCR replicates carried out for each sample. The number of parameters (K), model weights (w_i), and deviance are shown for each model and the models are ranked by their AICc differences relative to the best model in the set ($\Delta AICc_i$). Sigma (σ) was a random effect included in every model to account for unmodeled heterogeneity.
