# Supplementary material for: Sex and nest type influence avian blood parasite prevalence in a high-elevation bird community
Source: Parasit Vectors. 2021 Mar 8;14:145. doi: 10.1186/s13071-021-04612-w (PMC7938522; doi:10.1186/s13071-021-04612-w)
Supplement: Supplementary file 6 — Additional file 6: Table S6. Model rankings exploring factors affecting detection probability (p) and prevalence (ψ) of Haemoproteus parasites in Red-breasted Nuthatches. [file 13071_2021_4612_MOESM6_ESM.pdf]

Additional File 6 Model rankings exploring factors affecting detection probability (p) and prevalence (ψ) of *Haemoproteus* parasites in Red-breasted Nuthatches.

Model	K	ΔAICc	w_i	Deviance
σ(.) + p(.) + ψ(BCI)	3	0.00	0.69	31.50
σ(.) + p(.) + ψ(.)	2	2.21	0.24	36.42
σ(.) + p(.) + ψ(sex)	3	4.90	0.07	36.40

Model set and rankings exploring the importance of factors affecting the detection probability (p) and prevalence (ψ) of *Haemoproteus* blood parasites in Red-breasted Nuthatches captured and sampled at a high-elevation valley in northern Colorado during 2017-2018. 'PCR run' indicates the 3 PCR replicates carried out for each sample. The number of parameters (K), model weights (w_i), and deviance are shown for each model and the models are ranked by their AICc differences relative to the best model in the set ($\Delta AICc_i$). Sigma (σ) was a random effect included in every model to account for unmodeled heterogeneity.
