# Supplementary material for: Sex and nest type influence avian blood parasite prevalence in a high-elevation bird community
Source: Parasit Vectors. 2021 Mar 8;14:145. doi: 10.1186/s13071-021-04612-w (PMC7938522; doi:10.1186/s13071-021-04612-w)
Supplement: Supplementary file 8 — Additional file 8: Table S8. Model rankings exploring factors affecting detection probability (p) and prevalence (ψ) of Haemoproteus parasites in Dark-eyed Juncos. [file 13071_2021_4612_MOESM8_ESM.pdf]

Additional File 8 Model rankings exploring factors affecting detection probability (p) and prevalence (ψ) of *Haemoproteus* parasites in Dark-eyed Juncos.

Model	K	ΔAICc	w_i	Deviance
σ(.) + p(.) + ψ(.)	3	0.00	0.35	70.94
σ(.) + p(.) + ψ(sex)	4	1.32	0.18	69.61
σ(.) + p(PCR run) + ψ(.)	5	1.59	0.16	67.03
σ(.) + p(.) + ψ(BCI)	4	2.65	0.09	70.94
σ(.) + p(PCR run) + ψ(sex)	6	3.36	0.07	65.69
σ(.) + p(.) + ψ(sex+BCI)	5	3.96	0.05	69.39
σ(.) + p(PCR run) + ψ(BCI)	6	4.69	0.03	67.03
σ(.) + p(PCR run) + ψ(age)	6	6.21	0.02	68.54
σ(.) + p(PCR run) + ψ(sex+BCI)	7	6.51	0.01	65.47
σ(.) + p(.) + ψ(sex+age+BCI)	7	7.26	0.01	66.22
σ(.) + p(.) + ψ(sex+age)	7	8.43	0.01	67.39
σ(.) + p(.) + ψ(age+year+BCI)	7	8.74	0.00	67.70
σ(.) + p(.) + ψ(age+BCI)	7	9.30	0.00	68.26
σ(.) + p(PCR run) + ψ(age)	8	9.33	0.00	64.62
σ(.) + p(.) + ψ(sex+age+year+BCI)	9	11.03	0.00	62.30
σ(.) + p(PCR run) + ψ(sex+age)	9	12.22	0.00	63.48
σ(.) + p(PCR run) + ψ(age+BCI)	9	13.08	0.00	64.34
σ(.) + p(PCR run) + ψ(age+year)	7	14.40	0.00	73.37
σ(.) + p(PCR run) + ψ(sex+age+BCI)	10	15.45	0.00	62.28
σ(.) + p(PCR run) + ψ(sex+age+year)	8	15.97	0.00	71.26
σ(.) + p(PCR run) + ψ(sex+age+year+BCI)	11	16.39	0.00	58.33
σ(.) + p(PCR run) + ψ(year)	4	16.46	0.00	84.76
σ(.) + p(PCR run) + ψ(age+year+BCI)	10	16.96	0.00	63.79
σ(.) + p(PCR run) + ψ(sex+year)	5	18.47	0.00	83.90

σ(.) + p(PCR run) + ψ(year+BCI)	5	19.27	0.00	84.71
σ(.) + p(PCR run) + ψ(age+year)	9	19.36	0.00	70.63
σ(.) + p(PCR run) + ψ(year)	6	20.43	0.00	82.76
σ(.) + p(PCR run) + ψ(sex+year+BCI)	6	21.57	0.00	83.90
σ(.) + p(PCR run) + ψ(sex+age+year)	10	21.69	0.00	68.52
σ(.) + p(PCR run) + ψ(sex+year)	7	22.95	0.00	81.91
σ(.) + p(PCR run) + ψ(year+BCI)	7	23.75	0.00	82.71
σ(.) + p(PCR run) + ψ(sex+year+BCI)	8	26.62	0.00	81.91

Model set and rankings exploring the importance of factors affecting the detection probability (p) and prevalence (ψ) of *Haemoproteus* blood parasites in Dark-eyed Juncos captured and sampled at a high-elevation valley in northern Colorado during 2017-2018. 'PCR run' indicates the 3 PCR replicates carried out for each sample. The number of parameters (K), model weights (w_i), and deviance are shown for each model and the models are ranked by their AICc differences relative to the best model in the set ($\Delta AICc_i$). Sigma (σ) was a random effect included in every model to account for unmodeled heterogeneity.
