# Supplementary material for: Sex and nest type influence avian blood parasite prevalence in a high-elevation bird community
Source: Parasit Vectors. 2021 Mar 8;14:145. doi: 10.1186/s13071-021-04612-w (PMC7938522; doi:10.1186/s13071-021-04612-w)
Supplement: Supplementary file 9 — Additional file 9: Table S9. Model rankings exploring factors affecting detection probability (p) and prevalence (ψ) of Haemoproteus parasites in Warbling Vireos. [file 13071_2021_4612_MOESM9_ESM.pdf]

**Additional File 9** Model rankings exploring factors affecting detection probability (p) and prevalence (ψ) of *Haemoproteus* parasites in Warbling Vireos.

| Model | K | ΔAICc | $w_i$ | Deviance |
|---|---|---|---|---|
| σ(.) + p(.) + ψ(.) | 2 | 0.96 | 0.15 | 78.29 |
| σ(.) + p(PCR run) + ψ(BCI) | 5 | 1.33 | 0.13 | 69.54 |
| σ(.) + p(.) + ψ(BCI) | 3 | 1.54 | 0.12 | 76.16 |
| σ(.) + p(PCR run) + ψ(sex) | 5 | 1.63 | 0.11 | 69.85 |
| σ(.) + p(.) + ψ(sex) | 3 | 1.90 | 0.10 | 76.52 |
| σ(.) + p(.) + ψ(sex+BCI) | 4 | 2.79 | 0.06 | 74.40 |
| σ(.) + p(PCR run) + ψ(sex+BCI) | 6 | 3.29 | 0.05 | 67.65 |
| σ(.) + p(.) + ψ(age) | 4 | 6.48 | 0.01 | 78.09 |
| σ(.) + p(.) + ψ(age+BCI) | 6 | 6.65 | 0.01 | 71.01 |
| σ(.) + p(.) + ψ(age) | 6 | 7.04 | 0.01 | 71.40 |
| σ(.) + p(.) + ψ(sex+age) | 5 | 8.25 | 0.00 | 76.46 |
| σ(.) + p(PCR run) + ψ(age+BCI) | 7 | 8.27 | 0.00 | 68.23 |
| σ(.) + p(.) + ψ(sex+age+BCI) | 6 | 8.85 | 0.00 | 73.21 |
| σ(.) + p(PCR run) + ψ(sex+age) | 7 | 9.82 | 0.00 | 69.78 |
| σ(.) + p(PCR run) + ψ(sex+age+BCI) | 8 | 11.69 | 0.00 | 66.57 |

Model set and rankings exploring the importance of factors affecting the detection probability (p) and prevalence (ψ) of *Haemoproteus* blood parasites in Warbling Vireos captured and sampled at a high-elevation valley in northern Colorado during 2017-2018. ‘PCR run’ indicates the 3 PCR replicates carried out for each sample. The number of parameters (K), model weights ($w_i$), and deviance are shown for each model and the models are ranked by their AICc differences relative to the best model in the set (Δ**AICc**$_i$). Sigma (σ) was a random effect included in every model to account for unmodeled heterogeneity.
