# Supplementary material for: Sex and nest type influence avian blood parasite prevalence in a high-elevation bird community
Source: Parasit Vectors. 2021 Mar 8;14:145. doi: 10.1186/s13071-021-04612-w (PMC7938522; doi:10.1186/s13071-021-04612-w)
Supplement: Supplementary file 10 — Additional file 10: Table S10. Model rankings exploring factors affecting detection probability (p) and prevalence (ψ) of Haemoproteus parasites in White-crowned Sparrows. [file 13071_2021_4612_MOESM10_ESM.pdf]

Additional File 10 Model rankings exploring factors affecting detection probability (p) and prevalence (ψ) of *Haemoproteus* parasites in White-crowned Sparrows.

**Model**	**K**	**ΔAICc**	w_i	**Deviance**
σ(.) + p(.) + ψ(sex+BCI)	5	0.41	0.10	112.04
σ(.) + p(PCR run) + ψ(sex+year)	7	0.46	0.10	106.79
σ(.) + p(.) + ψ(sex+year)	5	0.86	0.08	112.48
σ(.) + p(PCR run) + ψ(sex)	6	1.17	0.07	110.21
σ(.) + p(PCR run) + ψ(year)	6	1.37	0.06	110.40
σ(.) + p(PCR PCR run) + ψ(.)	5	1.48	0.06	113.11
σ(.) + p(PCR run) + ψ(BCI)	6	1.51	0.06	110.55
σ(.) + p(.) + ψ(sex)	4	1.74	0.05	115.84
σ(.) + p(.) + ψ(year)	4	1.96	0.05	116.06
σ(.) + p(.) + ψ(BCI)	4	2.18	0.04	116.29
σ(.) + p(.) + ψ(.)	3	2.30	0.04	118.77
σ(.) + p(PCR run) + ψ(sex+year+BCI)	8	2.80	0.03	106.28
σ(.) + p(.) + ψ(sex+year+BCI)	6	2.95	0.03	111.98
σ(.) + p(.) + ψ(sex+age+BCI)	7	3.82	0.02	110.15
σ(.) + p(PCR run) + ψ(sex+age+BCI)	9	3.92	0.02	104.41
σ(.) + p(PCR run) + ψ(year+BCI)	7	4.03	0.02	110.36
σ(.) + p(.) + ψ(year+BCI)	5	4.39	0.01	116.02
σ(.) + p(.) + ψ(sex+age+year)	7	5.23	0.01	111.55
σ(.) + p(PCR run) + ψ(sex+age+year+BCI)	9	5.34	0.01	105.83
σ(.) + p(PCR run) + ψ(age)	7	5.42	0.01	105.83
σ(.) + p(.) + ψ(age)	5	5.78	0.01	117.40
σ(.) + p(PCR run) + ψ(sex+age)	8	5.87	0.01	109.34
σ(.) + p(.) + ψ(sex+age)	6	5.91	0.01	114.94
σ(.) + p(PCR run) + ψ(age+BCI)	8	6.58	0.00	110.06

σ(.) + p(PCR run) + ψ(age+year)	8	6.69	0.00	110.17
σ(.) + p(.) + ψ(age+BCI)	6	6.76	0.00	115.80
σ(.) + p(.) + ψ(age+year)	6	6.77	0.00	115.80
σ(.) + p(.) + ψ(sex+age+year+BCI)	8	7.50	0.00	110.98
σ(.) + p(PCR run) + ψ(sex+age+year+BCI)	10	7.91	0.00	105.26
σ(.) + p(.) + ψ(age+year+BCI)	7	9.43	0.00	115.75
σ(.) + p(PCR run) + ψ(age+year+BCI)	9	9.54	0.00	110.03

Model set and rankings exploring the importance of factors affecting the detection probability (p) and prevalence (ψ) of *Haemoproteus* blood parasites in White-crowned Sparrows captured and sampled at a high-elevation valley in northern Colorado during 2017-2018. 'PCR run' indicates the 3 PCR replicates carried out for each sample. The number of parameters (K), model weights (w_i), and deviance are shown for each model and the models are ranked by their AICc differences relative to the best model in the set (Δ**AICc**$_i$). Sigma (σ) was a random effect included in every model to account for unmodeled heterogeneity.
