# Supplementary material for: Sex and nest type influence avian blood parasite prevalence in a high-elevation bird community
Source: Parasit Vectors. 2021 Mar 8;14:145. doi: 10.1186/s13071-021-04612-w (PMC7938522; doi:10.1186/s13071-021-04612-w)
Supplement: Supplementary file 11 — Additional file 11: Table S11. Model rankings exploring factors affecting detection probability (p) and prevalence (ψ) of Haemoproteus parasites in Wilson’s Warblers. [file 13071_2021_4612_MOESM11_ESM.pdf]

Additional File 11 Model rankings exploring factors affecting detection probability (p) and prevalence (ψ) of *Haemoproteus* parasites in Wilson's Warblers.

Model	K	ΔAICc	w_i	Deviance
σ(.) + p(.) + ψ(sex+year)	5	0.01	0.19	101.85
σ(.) + p(.) + ψ(sex)	4	0.59	0.15	104.98
σ(.) + p(.) + ψ(year)	4	1.57	0.09	105.97
σ(.) + p(.) + ψ(BCI)	4	1.66	0.08	106.06
σ(.) + p(.) + ψ(.)	3	2.10	0.07	108.92
σ(.) + p(.) + ψ(sex+year+BCI)	6	2.69	0.05	101.84
σ(.) + p(.) + ψ(year+BCI)	5	4.09	0.03	105.93
σ(.) + p(PCR run) + ψ(sex+BCI)	7	4.89	0.02	101.20
σ(.) + p(PCR run) + ψ(sex+year)	7	4.90	0.02	101.21
σ(.) + p(PCR run) + ψ(sex)	6	5.19	0.01	104.35
σ(.) + p(.) + ψ(sex+age+BCI)	7	5.35	0.01	101.66
σ(.) + p(.) + ψ(sex+age+year)	7	5.38	0.01	101.69
σ(.) + p(.) + ψ(sex+age)	6	5.65	0.01	104.80
σ(.) + p(PCR run) + ψ(year)	6	6.18	0.01	105.33
σ(.) + p(PCR run) + ψ(BCI)	6	6.27	0.01	105.42
σ(.) + p(PCR run) + ψ(.)	5	6.44	0.01	108.28
σ(.) + p(.) + ψ(age+year)	6	6.77	0.01	105.92
σ(.) + p(.) + ψ(age+BCI)	6	6.85	0.01	106.01
σ(.) + p(.) + ψ(age)	5	6.94	0.01	108.78
σ(.) + p(PCR run) + ψ(sex+year+BCI)	8	7.89	0.00	101.19
σ(.) + p(.) + ψ(sex+age+year+BCI)	8	8.35	0.00	101.66
σ(.) + p(PCR run) + ψ(year+BCI)	7	8.98	0.00	105.29
σ(.) + p(.) + ψ(age+year+BCI)	7	9.59	0.00	105.90
σ(.) + p(PCR run) + ψ(sex+age)	7	10.85	0.00	104.16

σ(.) + p(PCR run) + ψ(sex+age+BCI)	9	10.88	0.00	101.01
σ(.) + p(PCR run) + ψ(sex+age+year)	9	10.92	0.00	101.05
σ(.) + p(PCR run) + ψ(age)	7	11.83	0.00	108.14
σ(.) + p(PCR run) + ψ(age+year)	8	11.97	0.00	105.28
σ(.) + p(PCR run) + ψ(age+BCI)	8	12.06	0.00	105.37
σ(.) + p(PCR run) + ψ(sex+age+year+BCI)	10	14.26	0.00	101.01
σ(.) + p(PCR run) + ψ(age+year+BCI)	9	15.13	0.00	105.26

Model set and rankings exploring the importance of factors affecting the detection probability (p) and prevalence (ψ) of *Haemoproteus* blood parasites in Wilson's Warblers captured and sampled at a high-elevation valley in northern Colorado during 2017-2018. 'PCR run' indicates the 3 PCR replicates carried out for each sample. The number of parameters (K), model weights (w_i), and deviance are shown for each model and the models are ranked by their AICc differences relative to the best model in the set (**ΔAICc**$_i$). Sigma (σ) was a random effect included in every model to account for unmodeled heterogeneity.
